# Epigenetic marks in the *Hyacinthus orientalis* L. mature pollen grain and during in vitro pollen tube growth

**DOI:** 10.1007/s00497-016-0289-3

**Published:** 2016-07-15

**Authors:** Marlena Kozłowska, Katarzyna Niedojadło, Marta Brzostek, Elżbieta Bednarska-Kozakiewicz

**Affiliations:** Department of Cell Biology, Faculty of Biology and Environment Protection, Nicolaus Copernicus University in Toruń, Toruń, Poland

**Keywords:** Epigenetic, 5mC, Histones, Pollen grain, Male germ unit, Reproduction

## Abstract

During the sexual reproduction of flowering plants, epigenetic control of gene expression and genome integrity by DNA methylation and histone modifications plays an important role in male gametogenesis. In this study, we compared the chromatin modification patterns of the generative, sperm cells and vegetative nuclei during *Hyacinthus orientalis* male gametophyte development. Changes in the spatial and temporal distribution of 5-methylcytosine, acetylated histone H4 and histone deacetylase indicated potential differences in the specific epigenetic state of all analysed cells, in both the mature cellular pollen grains and the in vitro growing pollen tubes. Interestingly, we observed unique localization of chromatin modifications in the area of the generative and the vegetative nuclei located near each other in the male germ unit, indicating the precise mechanisms of gene expression regulation in this region. We discuss the differences in the patterns of the epigenetic marks along with our previous reports of nuclear metabolism and changes in chromatin organization and activity in hyacinth male gametophyte cells. We also propose that this epigenetic status of the analysed nuclei is related to the different acquired fates and biological functions of these cells.

**Electronic supplementary material:**

The online version of this article (doi:10.1007/s00497-016-0289-3) contains supplementary material, which is available to authorized users.

## Introduction

In flowering plants, pollen grains germinate to form pollen tubes that transport the sperm cells to the egg cell in the embryo sac during sexual plant reproduction. During male gametogenesis, an asymmetric mitotic division of individual haploid microspores produces the smaller generative cell (GC), which is completely enclosed within the cytoplasm of a vegetative cell (VC). Depending on the plant species, the generative cell divides into two sperm cells (SCs), either during pollen maturation in the anther (tricellular pollen grain) or after pollination inside the pollen tube (bicellular pollen grain) (for review Twell 2011; Russell and Jones 2015). In most species, the generative cell, or one sperm cell, associates with the vegetative nucleus, establishing the ‘male germ unit’ (Russell and Cass 1981; Dumas et al. 1985; McCue et al. 2011). This physical connection allows it to travel as a unit through the growing pollen tube to the female gametophyte where fertilization of the egg and the central cell generates the embryo and the endosperm, respectively (McCue et al. 2011; Ge et al. 2011). Additionally, molecular data suggest that MGU linkage is essential for communication between the VC and SCs (Tian et al. 1998; Slotkin et al. 2009; Hamamura et al. 2011; Grant-Downton et al. 2013; Jiang et al. 2015).

Transcriptome (for review Rutley and Twell 2015) and translatome (Lin et al. 2014) studies of the male gametophyte indicated the dynamic changes in gene expression throughout development and in the specific cells. Generative and vegetative nuclei clearly show distinct transcriptional profiles supporting different functions (Borg et al. 2009; Baroux et al. 2011). SCs have their own unique patterns of transcription, their own promoters, cell cycle control factors and silencing elements (for review Russell and Jones 2015). Cell fate specification from genetically identical haploid cells is related to their dimorphic chromatin states. The chromatin of VCs is largely dispersed compared to the somatic cells, whereas the germline chromatin is maintained in a highly condensed state (She and Baroux 2014). Several studies suggest large-scale chromatin modifications associated with the epigenetic differentiation of gametophytic cells (Baroux et al. 2011). During male gametophyte development, there is a reprogramming of genome expression in which the dynamic events of DNA methylation, nucleosome remodelling and small RNA silencing take place. Cell-specific epigenome profiles are very relevant not only for the transcriptional state, but also for maintenance of the genome integrity of the male gametophyte (Slotkin et al. 2009; She and Baroux 2014; Fultz et al. 2015).

DNA methylation is an important mechanism for silencing transposons and other repetitive elements and for the stabilization of the genome by stable repression of specific transgenes and endogenous genes (Zhang and Zhu 2011). This process involves the addition of a methyl group to cytosine residues in DNA. Plants have at least three classes of cytosine methyltransferases maintained by three different pathways: CG methylation by DNA METHYLTRANSFERASE 1 (MET1), CHG methylation by CHROMOMETHYLASE 3 (CMT3) and asymmetric CHH methylation through persistent *de novo* methylation by DOMAINS REARRANGED METHYLTRANSFERASE 2 (DRM2), which act together with 24nt siRNA-based machinery (H = A, T, or C) (Law and Jacobsen 2010; Calarco et al. 2012; Movahedi et al. 2015). An important feature of epigenetic modifications is their reversibility. The cytosine methylation can be removed by the DNA glycosylases DEMETER (DME) (Zhu 2009; Schoft et al. 2011), REPRESSOR OF SILENCING 1 (ROS1) and DEMETER-LIKE 2 (DML2) and 3 (DML3) through a base excision repair mechanism (Law and Jacobsen 2010).

Genome-wide DNA methylation analysis of *Arabidopsis* indicated the dimorphic remodelling of DNA methylomes during male gametogenesis (She and Baroux 2014). The microspore chromatin is devoid of CHH methylation, mostly from retrotransposon loci, but retains CG methylation. After mitosis, the VN restores the CHH methylation of the transposable element (TE) loci and undergoes CG demethylation at a subset of TE loci and intergenic regions (Calarco et al. 2012). The chromatin remodelling factor, DECREASE IN DNA METHYLATION (DDM1), is down-regulated in the VN, with a concomitant increase in the transcripts of retrotransposons. TE-derived siRNAs generated in the VN are translocated to the sperm cells where they increase the silencing of the TEs in the gametic genome by reinforcing RdDM (Slotkin et al. 2009). Additionally, DME is restricted to the pollen VC, and it is possible that DME-induced hypomethylation of the TEs in the VN results in their transcriptional activation (Schoft et al. 2011; Gutierrez-Marcos and Dickinson 2012). Sperm cells inherit CHH DNA methylation, and CG methylation is globally retained. CHG methylation is higher in the VC, albeit depleted from the same demethylated CG TE loci (Calarco et al. 2012; Ibarra et al. 2012).

The composition of various histone modifications determines gene expression and the cellular state. In histone modification, lysines 9 and 27 of histone H3 can be either methylated or acetylated through chromatin remodelling. In general, H3K4 and H3K36 methylation correlates with transcriptional activation, whereas H3K9 and H3K27 methylation is associated with gene silencing (Ingouff and Berger 2010). Additionally, each lysine can be mono-, di- or tri-methylated, and these different methylation states can have distinct physiological effects. The homoeostatic balance of nucleosomal histone acetylation is maintained by the antagonic action of histone acetyltransferases (HAT) and histone deacetylases (HDAC) (Onder et al. 2012; Solís et al. 2012). Genetic analysis and cytological study revealed that post-translational histone modification dynamically changes during male gametophyte development. After asymmetric division of the microspore, the VC centromeric heterochromatin begins to decondense. This is accompanied by a progressive loss of cenH3, which persists in the centromeres of the germline (Ingouff et al. 2007; Schoft et al. 2009). Furthermore, SCs accumulate unique gamete-specific H3 variants between species, and they are completely removed from the zygote nucleus upon fertilization (Ingouff et al. 2007; Ingouff and Berger 2010). For example, *Arabidopsis* specifically accumulates the abundant male germline-specific variant H3.1 (Okada et al. 2005). Moreover, immunolocalization studies suggest that the highly condensed chromatin of SCs is preferentially enriched for active H3K4me2/me3 and H3K9ac marks, while H3K27me3 marks are specific to the VC (for review She and Baroux 2014; Borg and Berger 2015).

In this study, we have focused on the mechanism of chromatin activity regulation in the *Hyacinthus orientalis* male gametophyte. Using immunocytochemistry techniques, we examined the spatial and temporal distribution patterns of 5-methylcytosine (5mC), acetylated histone 4 (acH4), and histone deacetylase 1 (HDT1) in hyacinth bicellular pollen grain and in vitro grown pollen tubes. We discuss the differences in the localization of these epigenetic marks along with our previous reports (Zienkiewicz et al. 2006, 2008a, b, c, 2011) in which we examined nuclear metabolism and changes in the organization of the molecules involved in the key steps of gene expression in the VC, GC and SCs formed during pollen tube growth. We also propose that the epigenetic status of these cells is related to the change in acquired fate and biological function during male gametophyte development in this species.

## Materials and methods

### Plant material

*Hyacinthus orientalis* L. pollen grains and in vitro grown pollen tubes were used in the investigations. Freshly collected pollen grains were hydrated in a humid chamber for 30 min and then placed in Brewbaker and Kwak (1963) liquid medium containing 10 % (w/v) polyethylene glycol 4000 for germination and growth for 1.5–24 h. Cultivation was carried out at 26 °C in the dark. After three washes in PBS buffer at pH 7.2, unhydrated and hydrated pollen grains and the pollen tubes (after 1.5, 4, 6, 12, 14, 16, 18 and 24 h of cultivation) were fixed in a mixture of 4 % (w/v) paraformaldehyde (Polysciences), 0.25 % (v/v) glutaraldehyde (Sigma) prepared in PBS buffer at pH 7.2, overnight at 4 °C. Next, the material was washed in PBS buffer pH 7.2 and transferred to 0.01 M citrate buffer pH 4.8 and enzymatically digested in a mixture of 1.5 % cellulose R10 (Serva) and 20 % pectinase (Sigma-Aldrich) in 0.01 M citrate buffer pH 4.8 for 20 min at 35 °C. After washes in 0.01 M citrate buffer pH 4.8 and PBS pH 7.2, the protoplasts of the pollen grains and the pollen tubes were used for immunolabelling.

### Immunolocalization experiments

All immunocytochemical reactions using the material obtained in vitro were carried out in 1.5 ml Eppendorf tubes. The samples were gently centrifuged between each step of the procedure and placed on microscope slides covered with Biobond (BBI Solutions). 5-Methylcytosine was detected by incubating the material with a primary mouse anti-5mC antibody (Abcam, Cat. No. 73938) diluted 1:500 in PBS buffer pH 7.2 with 1 % BSA (Sigma) overnight at 4 °C and a secondary goat anti-mouse antibody Alexa Fluor 488 (Invitrogen) diluted 1:1000 in PBS buffer pH 7.2 with 1 % BSA for 1 h at 37 °C. For detection of acetylated histone H4, the material was incubated in a 5 % solution of powder milk for 10 min, washed in PBS buffer pH 7.2 and incubated with the primary rabbit anti-acH4 antibody (acetylated lysine 5, 8, 12, 16 on histone H4) (Agrisera, Cat. No. AS09 588) diluted 1:100 in PBS buffer pH 7.2 with 1 % BSA and 0.01 % Triton X-100 (Sigma) overnight at 4 °C and a secondary rat anti-rabbit antibody Alexa Fluor 488 (Invitrogen) diluted 1:1000 in PBS buffer pH 7.2 with 1 % BSA for 1 h at 37 °C. Histone deacetylase 1 (HDT1) was detected by incubating the material with a primary rabbit anti-HDT1 antibody (Agrisera, Cat. No. AS11 1792) diluted 1:250 in PBS buffer pH 7.2 with 1 % BSA overnight at 4 °C and a secondary rat anti-rabbit antibody Alexa Fluor 488 (Invitrogen) diluted 1:1000 in PBS buffer pH 7.2 with 1 % BSA for 1 h at 37 °C. Control reactions were performed without the primary antibodies. DNA was stained with DAPI (4′,6-diamidino-2-phenylindole) (Fluka). The samples were analysed with a Nikon Eclipse TE300 confocal laser scanning inverted microscope. The results were recorded using an argon-ion laser emitting light at a wavelength of 488 nm (blue excitation and green fluorescence). A 100 × (N.A. 1.3) Plan Fluor DIC H/N2 oil immersion lens was used. Images were collected in the green channel. The three-dimensional optical sections were acquired at 0.5 µm step intervals. The final images represent the projection of an image stack. The EZ 2000 Viewer software package (Nikon Europe BV, Badhoevedorp, The Netherlands) was used for image processing and analysis. An inverted Nikon Eclipse TE 80i fluorescence microscope, equipped with a mercury lamp, a UV-2EC UV narrow band filter, and a DXM 1200 FX digital camera, were used to visualize the DAPI staining.

### Neutral red staining

Hydrated pollen grains were suspended in germinating medium containing 0.001 % neutral red, and after 4 h of growth, the pollen tubes were collected. The DNA was stained with DAPI. The pollen tubes were analysed using an Olympus BX50 fluorescence microscope. The UPlanFI 100× (N.A. 1.3) oil immersion lens DIC H/N2 and narrow band filters (U-MNU, U-MNG) were used. The results were collected using an Olympus XC50 digital colour camera and Cell^B^ software (Olympus Soft Imaging Solutions GmbH, Münster, Germany).

### Quantitative evaluations

For quantitative measurements, each reaction step was performed using consistent values of temperatures, incubation times and concentrations of antibodies. The average fluorescence intensity from the nuclei was measured for 10 pollen grains or 10 pollen tubes from each developmental stage of three replicates. Only germinating pollen and pollen tubes with typical morphology of tip growth, e.g. cylindrical and regular shape were selected. The data were corrected for background autofluorescence as determined using the negative control signal intensities. The CeSa Statistical Analyser (Department of Cell Biology, Nicolaus Copernicus University, Toruń, Poland) software was used to evaluate the signal intensities. The signal intensity µm^−3^ was expressed in arbitrary units (a.u.) of the fluorescence intensity. Results of the quantitative data were analysed statistically using one-factor ANOVA and Newman–Keuls test. Analyses were performed using Statistica software (Statistica ver. 7, Statsoft). Figures are prepared in Microsoft Excel 2010.

## Results

**Fig. 1:**
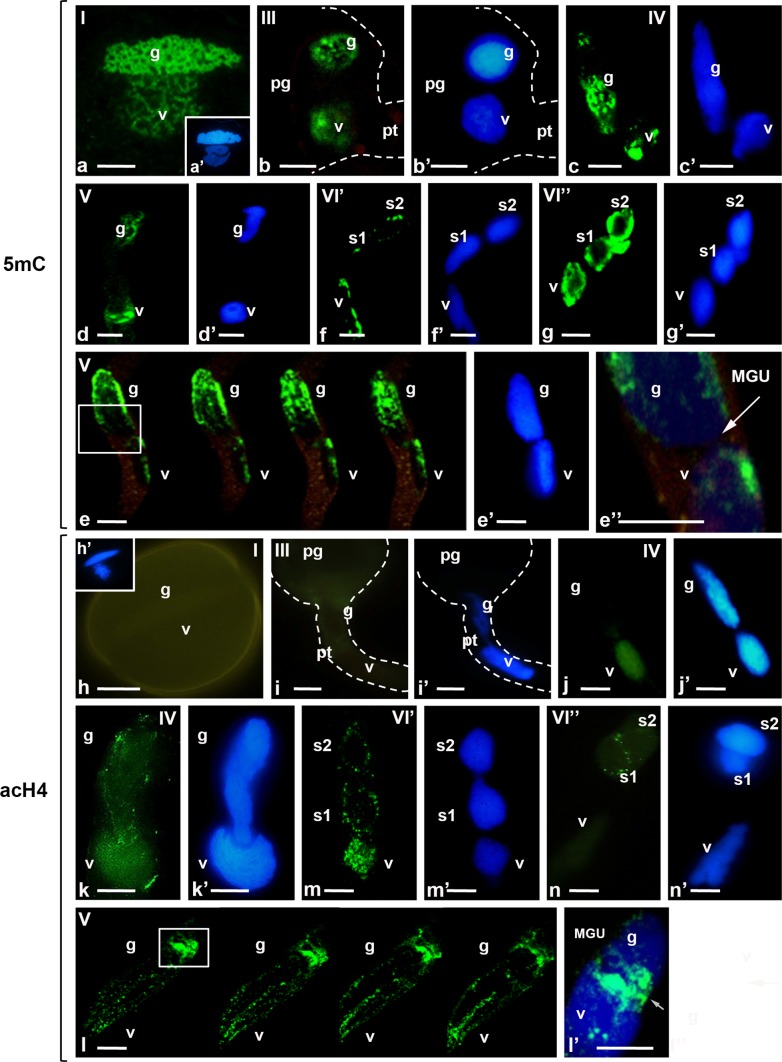
**a**–**g** Immunolocalization of 5mC (**a**–**g**) and acH4 (**h**–**n**) in the mature pollen grains and in vitro growing pollen tubes, **a**–**g** 5mC, **a** mature pollen grain (stage I), **b** germinating pollen grain (stage III), **c–f** the growing pollen tubes, **c** the early phase of growth (stage IV), **d** the late phase of growth (stage V), **e** serial optical sections of the MGU (stage V, after 6 h of cultivation)—the chromatin of the GN and the VN in adjacent areas of both nuclei of the MGU (*inset*), **e**′′ the magnification of the inset from **g** with the GN and the VN located near each other in the MGU (*arrow*), **f** pollen tube with sperm cells soon after the generative cell division (stage VI′), **g** pollen tube with sperm cells in late stage after the generative cell division (stage VI′′), **h**–**n** acH4 **h** mature pollen grain (stage I), **i** germinating pollen grain (stage III), **j**–**n** the growing pollen tubes, **j**, **k** the early stage of growth (stage IV), **l** serial optical sections of the MGU in the late phase of pollen tube growth (stage V), **l**′ the magnification of the inset from **l** with the area of the chromatin of both nuclei located close to each other (*arrow*), **m** pollen tube with sperm cells soon after the GC division (stage VI′), **n** pollen tube with sperm cells in the late growth stage after the GC division (stage VI′′), **a**′, **b**′, **c**′, **d**′, **e**′, **f**′, **g**′, **h**′, **i**′, **j**′, **k**′, **m**′, **n**′ DAPI staining, *g* generative nucleus, *v* vegetative nucleus, *MGU* male germ unit, *pg* pollen grain, *pt* pollen tube, *S1, S2* sperm cells, *scale bars* 10 μm

**Fig. 2:**
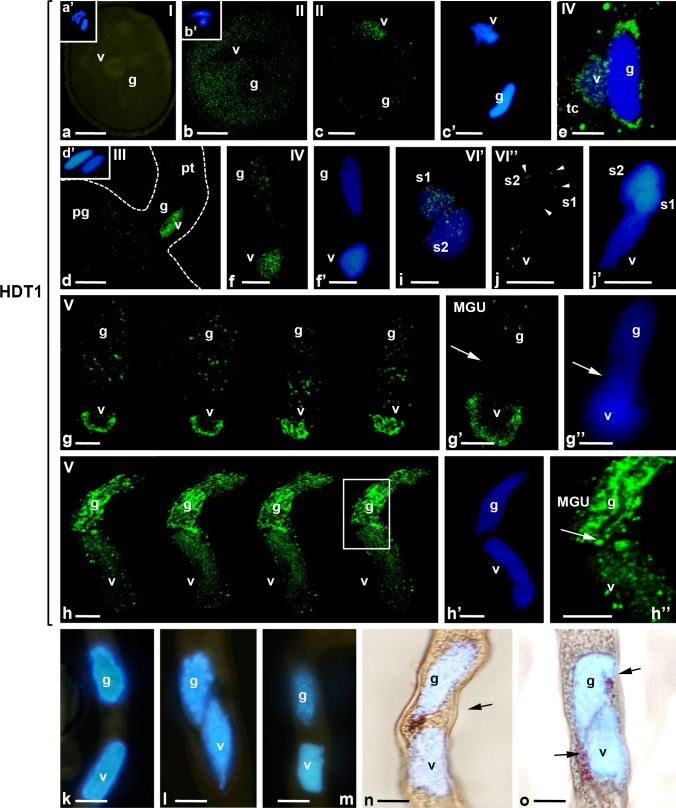
**a**–**j** Immunolocalization of HDT1 in the mature pollen grains and in vitro growing pollen tube, **a** mature pollen grain (stage I), **b**, **c** hydrated pollen grain (stage II), **d** the germinating pollen grain (stage III), **e**–**h** the growing pollen tube, **e**, **f** the early stage of growth (stage IV), **g**, **h** serial optical sections of the MGU with a different pattern of labelling, **g** pollen tube after 6 h of cultivation with single spots of fluorescence in the GN and a higher level of labelling in the VN, **g**′ no signal in the adjacent area of the GC and the VN (*arrow*), **h** pollen tube close to the GC division, **h**′′ the magnification of the VN and the GC located close to each other in MGU (*arrow*), **i** pollen tube with sperm cells soon after the GC division (stage VI′), **j** pollen tube with sperm cells in late phase after the GC division (stage VI′′), only a few spots of the fluorescence are visible in the nucleus of SC near the VN (*arrows*), **k**–**m** control reactions of 5mC **k**, acH4 **l**, HDC1 **m**–**o** MGU after neutral red staining, the physical relationship between the GC and the VN in the MGU is visible *(arrows)*, **a**′, **b**′, **c**′, **d**′, **f**′, **g**′′, **h**′, **j**′ DAPI staining, *g* generative nucleus, *v* vegetative nucleus, *tc* cytoplasm of pollen tube, *MGU* male germ unit, *pg* pollen grain, *pt* pollen tube, *S1, S2* sperm cells, *scale bars* 10 μm

Mature, dry pollen grains (stage I), pollen grains after 30 min. of hydration (stage II), and in vitro germinating and growing pollen tubes from *Hyacinthus orientalis* were used in the investigation. In the hours following cultivation, the pollen tubes had different lengths, indicating their physiological diversity. Therefore, we divided this material into five additional stages. Stage III consists of the germinating pollen grains when the VN and the GC have moved from the pollen grain into the pollen tube. The early phase of pollen tube growth, during 1.5–4 h of cultivation, when the pollen grains contain a large vacuole and the vacuoles also occur in the pollen tubes is labelled as stage IV. Stage V is the late phase of pollen tube growth, between 6 and 12 h of cultivation, when the pollen tubes form callose plugs and the cytoplasm is localized near the growing tip. When the pollen tubes have SCs (after the GC division), 14–16 h of cultivation is stage VI′, and 18–24 h of cultivation is called stage VI′′. We analysed the patterns of the distribution of the examined epigenetic marks in the nuclei of the male gametophyte in each of the described stages from mature pollen grain to the formation of the SCs (Fig. 1, 2, Supplementary material 2).

The GC of *H. orientalis* contains the characteristic vesicles of the reticular origin (coloured bodies), which are visible after vital neutral red staining (Bednarska 1988). This allows for the observation of the GC’s cytoplasm in the growing pollen tube. Simultaneous localization of the nuclei by DAPI staining and the cytoplasm of the GC by neutral red staining revealed the presence of the male germ unit (MGU). The cytoplasmic projection of the spindle-shaped GC is localized near the VN (Fig. 2n, o), and the effect of the spatial organization of these structures is the physical relationship between the GC and VN (Fig. 2n, o, arrows). Therefore, in our studies, we focused on the patterns of the analysed epigenetic marks in the organized structures in the areas in which the chromatins of the VN and the GN are located close to each other.

### Immunolocalization of 5mC in pollen grain and during pollen tube growth

**Fig. 3:**
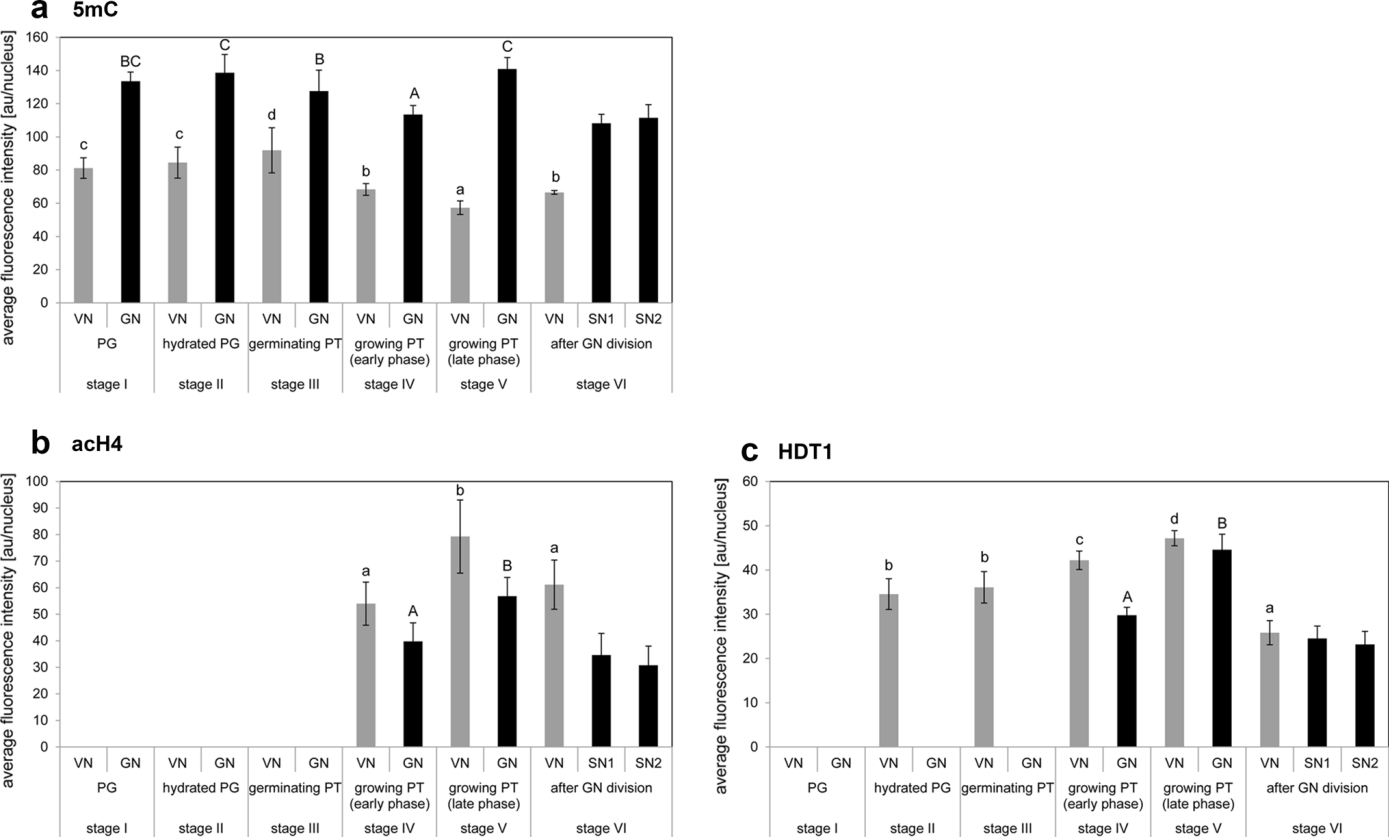
Quantitative analysis of the 5mC **a**, acH4 **b** and HDT1 **c** fluorescence in dry, mature pollen grain (stage I), hydrated pollen grain (stage II), germinating pollen tubes (up to 4 h of cultivation, stage III), the early phase of pollen tube growth (4–12 h of cultivation, stage IV), the late phase of pollen tube growth (stage V), and the pollen tube after the GC division (12–14 h of cultivation and longer). Relative fluorescence intensity is given as arbitrary units (a.u.) per nucleus (mean ± standard deviation). Values marked with different letters (*lower case letters* for VN,* capital letters* for GN) differ significantly (*p* < 0.05). *PG* pollen grain, *PT* pollen tube, *VN* vegetative nucleus (*grey square*), *GN* generative nucleus (*black square*)

Measurements of average fluorescence intensity confirmed the significant differences in the levels of 5mC within the chromatin region of male gametophyte nuclei (Fig. 3a). In the pollen grains, we localized 5mC in both the GN and the VN chromatin (Fig. 1a). In the mature dry pollen grain (stage I), the fluorescence signal, which reflected the chromatin organization, of the highly condensed chromatin of the GC was higher than that of the less condensed chromatin of the VN (Fig. 3a). After hydration (stage II) and during the germination (stage III) of the pollen tubes, the high 5mC labelling was visible further in the nuclei of both pollen cells. However, the patterns of the fluorescence of the chromatins were more dispersed (Fig. 1b). In the VN, we mainly observed diffuse labelling, whereas in the GN, small fluorescent spots were detected, in addition to the diffuse signal. During early pollen tube growth phase (stage IV), a gradual decrease of the 5mC labelling occurred in the VN and the GN (Fig. 1c). The areas of nucleus devoid of the fluorescence were localized mainly in the VN, and the signal of 5mC labelling was observed in large aggregates. Additionally, the signal of the fluorescence in the GN was still observed. Despite the change in the distribution pattern of 5mC, the average fluorescence level of VN and GN chromatin remained at a similar level (Fig. 3a). During the late phase of pollen tube growth (6–12 h, stage V), we observed a slight decrease in the fluorescence in the VN, while an increase in the fluorescence level in the GN (Figs. 1d, 3a). The analysis of the optical serial sections of the VN and the GN organized in an MGU in pollen tubes revealed that the chromatin of the GN and the VN within their vicinity had lower levels of the fluorescence or no detectable signal (Fig. 1e, inset, Fig. 1e′′, arrow). After GC division (stage VI′), the average level of signal in VN remained at a similar level, and the signal level was high in the SNs (Fig. 3a). The nuclei of both the VN and the SCs still contained areas devoid of the fluorescent signal which appeared in the form of spots (Fig. 1f). The analysis of the distribution of 5mC indicates that at this stage of pollen tube growth, increasing DNA methylation in both the VN and the SNs can be observed (compare Fig. 1d, f). In older cultures (stage VI′′), strong uniform fluorescence indicating the presence of 5mC was located in the VN and the SCs nuclei (Fig. 1g).

### Distribution of acetylated histone H4 in pollen grain and during pollen tube growth

Quantitative analysis confirmed the significant differences in the levels of acH4 signal in male gametophyte nuclei during studied stages of development (Fig. 3b). In the dry and hydrated pollen grains (stage I–II) in the GN and the VN, we did not observe the fluorescent signal indicating the presence of acH4, which is the marker of active chromatin (Fig. 1h). Additionally, during the initial germination of the pollen grains when the VN and the GN move to the pollen tube (stage III), we did not detect the acH4 labelling signal in either nuclei (Fig. 1i). The signal indicating the presence of acH4 did not appear until during early pollen tube growth. Initially, the fluorescence was only present in the VN (Fig. 1j). In the longer pollen tubes, after 4 h of growth (stage IV), the labelling was also observed in the GN (Fig. 1k). During this period of pollen tube growth, the diffuse signal was mostly visible in the VN, but we also observed small fluorescent spots localized mainly in the periphery of the chromatin in the GN. The fluorescence level of acH4 was slightly higher in the VN compared to the GN (Fig. 3b). During the late phase of pollen tube growth (stage V), the fluorescent signal indicating acH4 was present in both cells and formed aggregates (Fig. 1l). However, it was still higher in the VN than in the GN (Fig. 3b). Using confocal microscopy, we observed the VN and the GN located close to each other in the MGU (Fig. 1l). In the chromatin, we observed increased acH4 signal in the adjacent areas of both the nuclei (Fig. 1l′, arrow). After the GC division (stage VI), the signal from acH4 gradually decreased in both the vegetative and sperm nuclei (Fig. 1m, n). Just after the GC division (stage VI′), the fluorescent spots were still present in the VN, and a single spot of the signal was also localized in the nuclei of the SCs (Fig. 1m). The fluorescent spots were arranged mainly in the periphery of the chromatin. In the older cultivars (18–24 h, stage VI′′) of the pollen tubes with SCs, we did not observed fluorescence in the VN (Fig. 1n). It was surprising that in this stage of the male gametophyte development, we detected a different pattern of acH4 localization in the SC nuclei. Generally, a single focus of fluorescence was present in the periphery of the chromatin only in the SC near the VN (Fig. 1n).

### Levels of HDT1 in pollen grain and during pollen tube growth

Quantitative analysis confirmed the significant differences in the distribution of HDT1 in the chromatin of male gametophyte nuclei (Fig. 3c). In the VN and the GN of the dry pollen grains (stage I), we did not observe the signal for HDT1 (Fig. 2a). However, in pollen grains after hydration (stage II), the fluorescence signal was observed in the VC. In this cell, two labelling patterns were detected. In the first, the numerous small foci of fluorescence were visible only in the cytoplasm (Fig. 2b). In the second, the number of fluorescent spots in the cytoplasm of the VC was lower, and the signal of HDT1 was present mainly in the nucleus (Fig. 2c). During germination of the pollen grains (stage III), we observed increased fluorescence in the VN, and a single focus of the signal was still visible in the cytoplasm of this cell (Fig. 2d). In these samples, labelling was not observed in the GC. After the movement of the MGU to the pollen tube, the HDT1 signal was observed in the cytoplasm of the GC (Fig. 2e). During the early phase of pollen tube growth (stage IV) in the pollen tube and the GC, the cytoplasmic signal decreased, while the pool of HDT1 in the nucleus increased (Fig. 2f). During the entire period of pollen tube growth, until the GC division, there was a progressive increase in the level of HDT1 in both the VN and the GN (Fig. 3c). However, the level of this marker remained higher in the VN than in the GN, where we observed only single spots of the signal. This labelling pattern was still present during the late phase of pollen tube growth (stage V) (Fig. 2g). The serial optical sections of the MGU showed that a small focus of the fluorescence that was present in the GN was dispersed, while in the VN, the signal was more aggregated. The chromatin located in the region of spatial proximity between the VN and the GN nuclei in the MGU was devoid of the signal indicating the presence of HDT1 (Fig. 2g′, arrow). Just before the GC division (12 h of cultivation) in the GN, a strong HDT1 signal was localized in the highly condensed chromatin fibrils, while in the VN, the signal was visualized at the periphery of the chromatin in a dispersed pattern of small foci (Fig. 2h). The analysis of the serial optical sections of the MGU revealed that during this period of pollen tube growth, the signal was present in vegetative and generative chromatin in the adjacent areas of the VN and the GN (Fig. 2h′′, arrow). Soon after the GC division (stage VI′), the HDT1 signal in small numerous foci was seen in the VN (not shown) and in the SC nuclei (Fig. 2i). During further cultivation, the amount and the dimension of the signal spots in the VN and the SC nuclei were decreased. In 24 h of cultivation (stage VI′′), we localized only a few small spots of the fluorescence in the VN. During this cultivation period, we only observed small numerous foci in one nucleus of the SCs near the VN (Fig. 2j, arrows).

In the control reactions, which used no primary antibodies against 5mC (Fig. 2k), acH4 (Fig. 2l) or HDT1 (Fig. 2m), there was no labelling in the cells of the male gametophyte.

## Discussion

**Fig. 4:**
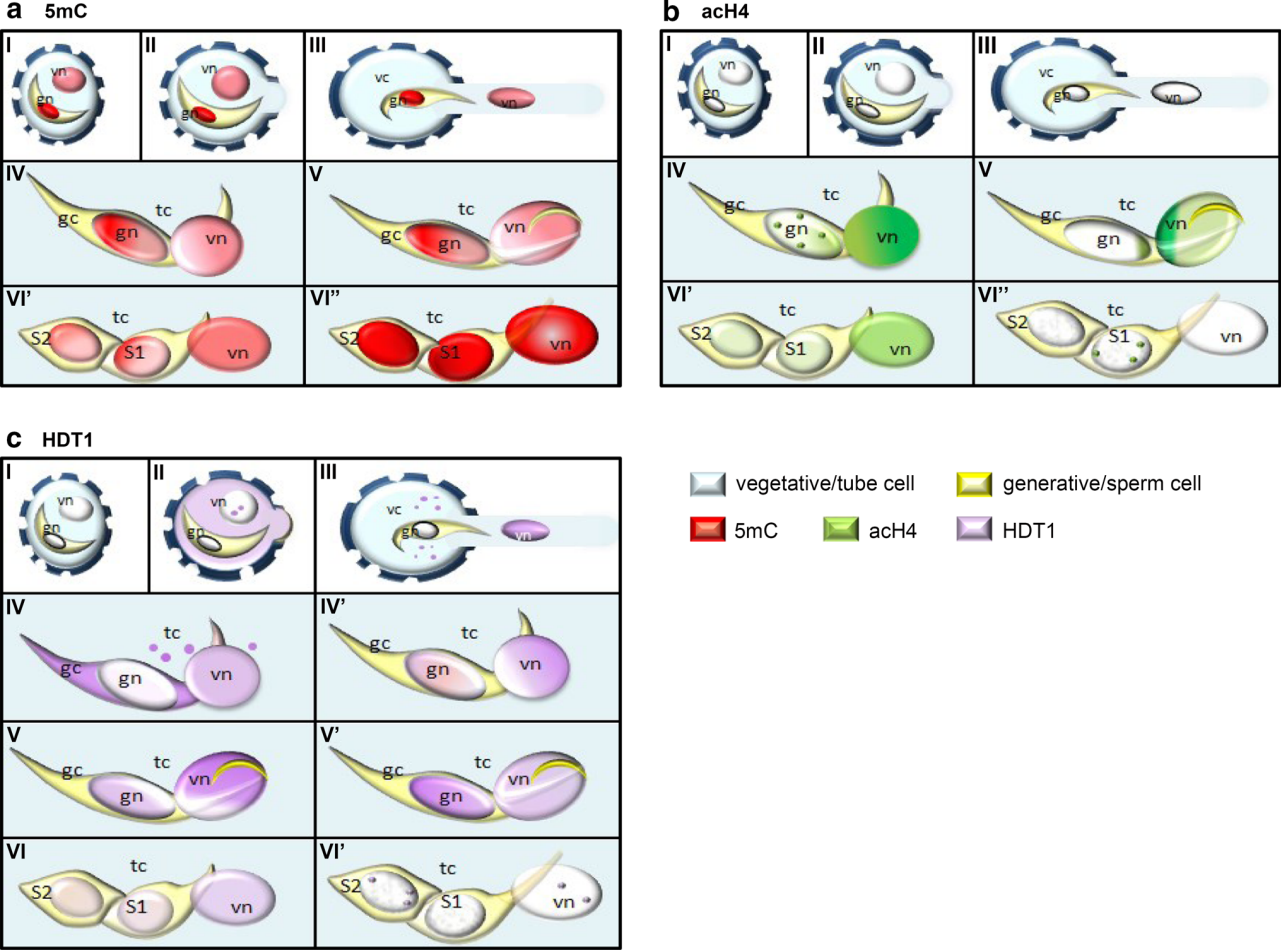
The distribution of the epigenetic marks in the *Hyacinthus orientalis* pollen grain and in vitro growing pollen tube, **a** in the mature pollen grain and in the growing pollen tube, the strongly methylated DNA was observed in the GN rather than the VN (stage I*–*V); after the GC division, the increased level of 5mC in both the VN and the SNs was visible (stage VI′*–*VI′′), **b** after the GC and the VN move to the pollen tube, acH4 was localized in the chromatin of both nuclei (stage IV*–*V), the labelling of the VC was higher than in the GC, the decreasing level of acH4 fluorescence after the GC division was observed in the SNs until it completely disappeared in the VN (stage VI′*–*V′′), **c** after the hydration of the pollen grain, HDT1 was present in the cytoplasm and in the nucleus of the VC (stage II–III); in the GC, the enzyme was localized after the move to the pollen tube, first in the cytoplasm and then in the nucleus (stage IV); during pollen tube growth, HDT1 was present in both nuclei, but the level was higher in the VN than in the GC (stage IV, IV′–V); before mitosis, the increase of the HDT1 signal was localized to the GC (stage V′); after the GC division, the level of HDT1 in both the VN and in the SNs was decreased (stage VI′′–VI′′), *vn* vegetative nucleus, *vc* cytoplasm of vegetative cell, *gn* generative nucleus, *gc* cytoplasm of generative cell, *tc* cytoplasm of pollen tube, *S1, S2* sperm cells

Our studies revealed that during the growth of the pollen tube, there are dynamic changes in the distribution pattern and in the level of the epigenetic marks on the chromatin of the *H. orientalis* male gametophyte cells (summary in Fig. 4). In mature, dry pollen grains, the higher level of 5mC in the GN than in the VN appears to confirm that the GN has a more condensed chromatin state than the chromatin of the VN (Bednarska and Górska-Brylass 1987). In these nuclei, however, neither euchromatin marker acH4 nor the HDT1 enzyme, which removes acetyl residues, was found. High levels of DNA methylation in the nuclei of the mature pollen grain have been previously observed in other species (Oakeley et al. 1997; Janousek et al. 2000; Ribeiro et al. 2009; Houben et al. 2011). In *Lilium longiflorum*, a species closely related to hyacinth, DNA hypermethylation is accompanied by H4 deacetylation during pollen development (Janousek et al. 2000). It can therefore be proposed that high levels of DNA methylation with H4 hypoacetylation are an important mechanism for temporarily blocking the transcriptional activity during the period of pollen quiescence (Zienkiewicz et al. 2006, 2008b, c). This ensures the survival of the male gametophytes during the period of time without an external source of nutrients from anthesis to pollination until they reach the stigma.

Shortly after rehydration of the pollen grain in *H. orientalis* in the nuclei of both cells, especially in the VC, dispersion of the 5mC signal was visible. This probably results from the reorganization of the chromatin structure, which becomes largely decondensed. It primarily concerns the VN, which is prepared to restart RNA synthesis during pollen tube growth (Zienkiewicz et al. 2008a, b; Lenartowski et al. 2014). However, in this period of pollen development, acH4 was not observed in either the VN or the GN. Such a distribution pattern indicates that their chromatin remains inactive. Surprisingly, despite the lack of acH4, in the VC, we localized the HDT1 enzyme—first in its cytoplasm and then also in the nucleus. This reveals that during the hydration of the pollen in VC, *de novo* synthesis of HDT1 occurs, which is then exported to the nucleus prior to the histones’ acetylation (Liu et al. 2014). It is known that in mature pollen, there is a pool of transcripts for the production of proteins associated with the first period of germination of the pollen tube, e.g. rearrangement of the cell wall and the cytoskeleton, as well as transcripts coding transcription factors involved in the regulation of gene expression in the subsequent stages of pollen tube growth (Mascarenhas 1990; Honys and Twell 2003, 2004; Becker et al. 2003; Ishimizu et al. 2010; Rutley and Twell 2015). The obtained results thus indicate that in the mature *H. orientalis* pollen grain, one can find transcripts encoding enzymes associated with the regulation of histone acetylation in the pool of long-lived mRNAs (Zienkiewicz et al. 2006). Rapid transport of these proteins into the nucleus, in the absence of acetylated histone variants, may suggest that the enzyme is either stored in an inactive form or it is necessary to protect the chromatin against its premature activation. In the GC, the HDT1 enzyme also appears to be synthesized *de novo*, however, later than in the VC. We visualized it in the cytoplasm of this cell only after its movement to the pollen tube. However, in the VC and the GC, HDT1 synthesis precedes the appearance of acH4 in the chromatin and occurs before restarting its transcriptional activity, which was observed after several hours of cultivation (Zienkiewicz et al. 2011). The study of the transcriptome of pollen cells in *A. thaliana*, rice, tobacco and others (for review Rutley and Twell 2015) has demonstrated that in the GC, numerous transcripts are present, and those encoding HDT1 are probably among them.

After germination of the pollen and during the early stages of pollen tube growth in hyacinth decreasing the level of DNA methylation with the simultaneous appearance of acetylated histones in the VN, and later in the GN, correlates with the period of restarting transcriptional activity (Zienkiewicz et al. 2008a, 2011). Such a DNA demethylation process, as well as an increase of H4 acetylation in the VN, has already been observed in the *Quercus suber* pollen grain (Ribeiro et al. 2009) and during *Lilium longiflorum* pollen tube growth (Janousek et al. 2000). From studies involving other cellular models, it is known that histone acetylation decreases the interactions between neighbouring nucleosomes and prevents their compaction, leading to a looser structure of the chromatin (Görish et al. 2005; Shahbazian and Grunstein 2007). This more ‘open’ structure of the chromatin facilitates binding of transcription factor complexes to promoter regions and leads to the activation of gene transcription (Niedojadło et al. 2012; Ma et al. 2013). Therefore, it can be suggested that the localization of acH4 can be found in hypomethylated regions of DNA, which occurs in the gametophyte nuclei during pollen tube growth.

Methylated cytosine also plays an important role in maintaining the integrity and the stability of the genome of the gametes and protecting the genome against transposons. Schoft et al. (2011) revealed the expression of the DEMETER (DME) glycosylase and their homologues, ROS1, DEMETER-LIKE2 (DML2) and DML3, in the VN of the *A. thaliana* pollen grain. None of these genes was found to be expressed in the sperm cells. It is possible that DME-induced hypomethylation of the TEs in the VN results in their transcriptional activation. This may also contribute to an increase in TE-derived siRNAs, which accumulate in sperm cells and enhance TE silencing via RNA-directed DNA methylation (RdDM) (Slotkin et al. 2009; Calarco et al. 2012). Reduction in the level of 5mC and the epigenetic reorganization of the chromatin in the VN in the absence of replication in hyacinth **(**Bednarska and Górska-Brylass 1987) also suggests an active mechanism of DNA demethylation, but more detailed studies are needed to address this hypothesis.

The restarting of gene expression requires the presence of active RNA pol II. We have previously demonstrated that in nuclei of mature *H. orientalis* pollen, despite transcription inhibition, the inactive form of RNA pol II (Pol IIA) is present, while in growing pollen tubes, the elongation form of this enzyme (Pol IIO) is found (Zienkiewicz et al. 2008a, b, 2011). Recent studies of the regulation of RNA pol II have shown that histone acetylation accelerates the transition of RNA pol II from the initiation to the elongation form (Stasevich et al. 2014). One cannot exclude that a similar process occurs during the restarting of transcription in the growing pollen tube. During pollen tube growth, not only the level of acH4, but also its distribution pattern in the VN and the GN was different. In the VN, this active chromatin mark was uniformly distributed throughout the nucleus, indicating that the chromatin of the VN more globally transforms into euchromatin. However, in the GN, acH4 was located mainly in the clusters and at the periphery of the nucleus. Thus, we can speculate that clusters of acetylated histones identify the euchromatin regions in which genes specifically expressed in this cell are located. This pattern of acH4 distribution is in agreement with previous observations. In the growing pollen tube of *H. orientalis,* transcriptional activity of the VN is higher than that observed in the GN (Zienkiewicz et al. 2011). Additionally, at later stages of pollen tube growth in the VN, the acH4 distribution pattern becomes more clustered, while in the GN, shortly before its division, acH4 levels decrease significantly. These differences probably reflect the changes occurring in the chromatin of these nuclei and are probably associated with a reduction of transcriptional activity.

In our studies, we are concerned with the specific regions of the VN and the GN that are close to each other in the MGU. Dynamic changes of the chromatin epigenetic marks indicate that there may be some euchromatin regions where the genes expressed specifically in the pollen tube and the GC are localized and they may constitute a mechanism of precise regulation of their expression. The MGU, composed of the VN and its associated GC/SCs, was described as a functional unit as early as the 1980s in species producing tricellular (Russell and Cass 1981; Dumas et al. 1985; McConchie et al. 1985; Mogensen 1992) and bicellular pollen grains (Wagner and Mogensen 1988; Tian et al. 1998). Presently, this unique structure is more often defined as a physiological unit (Russell et al. 2010; Ge et al. 2011; McCue et al. 2011). Recently, it has been speculated that the presence of ‘plasmodesmata-like’ canals, which link the GC and the VN cytoplasm (Cresti et al. 1987), may facilitate communication and transport between these cells. In turn, numerous nuclear pores on the envelope of the VN and the GN in the region of their proximity (Yu et al. 1989; McCue et al. 2011) might suggest that nuclear molecules are also transported (Slotkin et al. 2009, Jiang et al. 2015).

The increase of the level of HDT1 in the VN and the GN was observed shortly before the formation of sperm cells. The larger pool of HDT1 in the studied nuclei seems to correlate with decreasing acH4 euchromatin. Thus, we are convinced that it is one of the mechanisms involved in the inhibition of transcriptional activity of the vegetative and generative nuclei, which occurs during this period of pollen tube growth (Zienkiewicz et al. 2008a, b, 2011). Increases to the pool of HDT1 were particularly evident in the GN, as it prepares for the division. In the *A. thaliana* GC shortly before its division, *DUO1* (DUO POLLEN 1) is transcribed. *DUO1* encodes a transcription factor and plays a key role in sperm cell formation by activating expression of germline genes (Brownfield and Twell 2009; Brownfield et al. 2009). DUO1 is activated by the ARID1 protein (AT-Rich Interacting Domain), which physically associates with histone deacetylase 8 (HDA8) and facilitates the maintenance of histone acetylation in pollen (Zheng et al. 2014). In addition, it is known that the cascade of histone modification, including deacetylation of histone H4 lysine 16, is involved in the formation of mitotic chromosomes. Removal an acetyl group promotes the freeing of the H4 tail to interact with the surface of neighbouring nucleosomes and fibre condensation (Wilkins et al. 2014). Given all these facts, we postulate that HDT1 in the GN may also participate in the preparation for mitosis.

In *H. orientalis* pollen tubes with SCs, the VN as well as the gametes’ nuclei exhibit low transcriptional activity for a few hours after mitosis II (Zienkiewicz et al. 2011). Our data indicate that the ‘maturation’ of the gametes was accompanied by an increase in DNA methylation and a decrease in the pool of acH4 and HDT1. Surprisingly, we often observed a diversity of SCs showing the presence of acH4 and HDT1. During the period in which the VN was deprived of acH4, single spots of this euchromatin mark were present on the borders of the sperm chromatin. These spots only occurred in the gamete that remained in contact with the VN. Interestingly, we did not localize HDT1 in the nucleus of the gamete. This may indicate that the chromatin of ready-to-fertilize *H. orientalis* male gametes is epigenetically diverse. Preferential fertilization in species with morphologically diverse gametes has been previously described (Russell 1984, 1985; Yu et al. 1992; Gou et al. 2009). Whether the presence of euchromatin regions differentiates the gametes remains an open question, which requires further precise investigations. At this stage of pollen tube growth, there is progressive DNA methylation in the VN, accompanied by a decrease in the pool of acH4 until it completely disappears. This reveals the heterochromatization of the nucleus, which is well known to be degraded after fulfilling its biological function.

In conclusion, our in situ studies indicate the presence of epigenetic mechanisms related to the organization of the chromatin of hyacinth male gametophyte cells. These epigenetic mechanisms also have an effect on the processes associated with the regulation of gene expression as well as the maintenance of the stability and the integrity of the gametes’ genomes. Spatiotemporal changes of 5mC, acH4 and HDT1 distribution suggest a potentially different epigenetic status of the vegetative, generative and sperm cells, which is closely related to their functions. Simultaneous presence of eu- and heterochromatin marks indicates precise and complex processes of reprogramming the male gametophyte cells, the proper course of which determines the success of double fertilization and embryogenesis.

### **Author contribution statement**

K. N. and E. B. K. conceived and designed the experiments; M. K., K. N. and M. B. performed the experiments; M. K., K. N. and E. B. K. analysed the data and wrote the paper.

## Electronic supplementary material

Below is the link to the electronic supplementary material.

Supplementary material 1 (DOCX 14 kb)

Supplementary material 2 (PDF 51317 kb)

Supplementary material 3 (DOCX 24 kb)

## Abbreviations

MGU: Male germ unit
VC: Vegetative cell
VN: Vegetative nucleus
GC: Generative cell
GN: Generative nucleus
SC: Sperm cell
5mC: 5-Methylcytosine
HDT1: Histone deacetylase 1
acH4: Acetylated histone H4
