# Supplementary material for: Epigenetic marks in the Hyacinthus orientalis L. mature pollen grain and during in vitro pollen tube growth
Source: Plant Reprod. 2016 Jul 15;29:251–63. doi: 10.1007/s00497-016-0289-3 (PMC4978762; doi:10.1007/s00497-016-0289-3)
Supplement: Supplementary file 1 — Supplementary material 1 (DOCX 14 kb) [file 497_2016_289_MOESM1_ESM.docx]

Supplementary material 1. Different localization pattern of the epigenetic marks in the *Hyacinthus orientalis* pollen grains and *in vitr*o growing pollen tubes:

Fig. S1 Immunolocalization of 5mC in *in vitro* growing pollen tubes in **a –f** the early phase of growth (stage IV) and **g – j** (**j, j’, j’’**- serial optical sections) the late phase of growth
(stage V), **a’, b’, c’, d’, e’, f’, g’, j’’’** DAPI staining

Fig. S2 Localization pattern of acH4 in **a** the hydrated pollen grain (stage II) and *in vitro* growing pollen tubes **b - d** in the early phase of growth (stage IV) and **e – g** in the late phase of growth (stage V), **a’, b’, c’, d’, e’, f’, g’** DAPI staining

Fig. S3 Immunolocalization of HDT1 in **a - b** the hydrated pollen grain (stage II) (**b’** – pow the generative nucleus, **b’’’** pow the vegetative nucleus from **b**), **c- e** the germinating pollen tubes (stage III) and *in vitro* growing pollen tubes **f** in the early phase of growth (stage IV) and **g** in the late phase of growth (stage V), **a’, b’, c’, d’, e’, f’, g’** DAPI staining

Fig. S4 Localization pattern of HDT1 in **a – d** *in vitro* growing pollen tubes in the early phase of growth (stage IV), **c-c’’’** and **d – d’’** - serial optical sections, **a’, b’, c’’’’, d’’’** DAPI staining

Fig. S5 Localization pattern of the epigenetic marks in *in vitro* growing pollen tubes with sperm cells (stage VI), **a** 5mC, **b – c** acH4, **d** HDT1, **a’, b’, c’, d’** DAPI staining

*g* – generative nucleus, *v* – vegetative nucleus, *MGU* – male germ unit, *pg* – pollen grain, *pt* – pollen tube, *S1, S2* – sperm cells, Scale Bars 10 μm
