# Supplementary figures and images for: Epigenetic marks in the Hyacinthus orientalis L. mature pollen grain and during in vitro pollen tube growth
Source: Plant Reprod. 2016 Jul 15;29:251–63. doi: 10.1007/s00497-016-0289-3 (PMC4978762; doi:10.1007/s00497-016-0289-3)

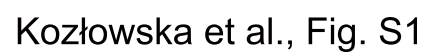

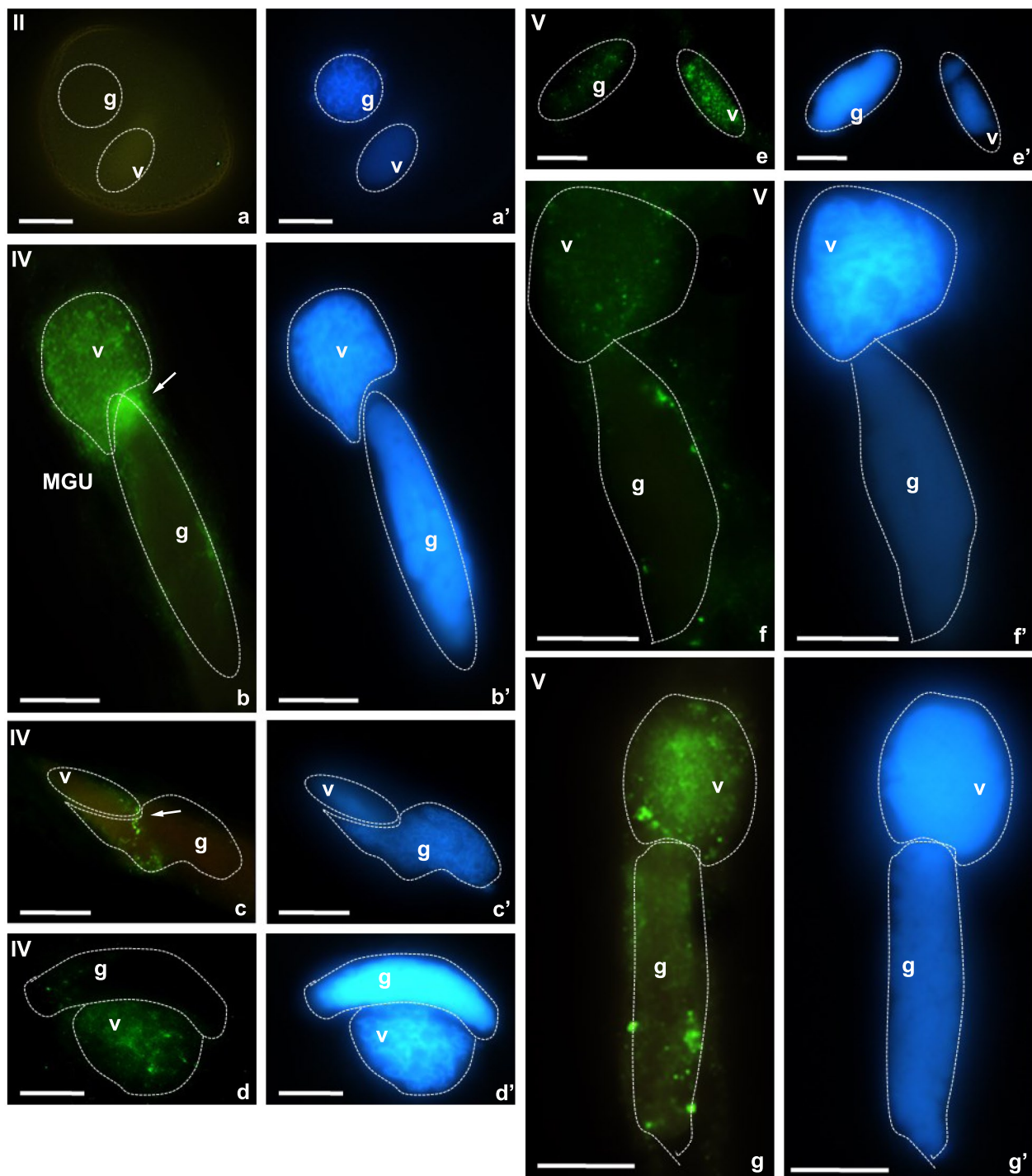

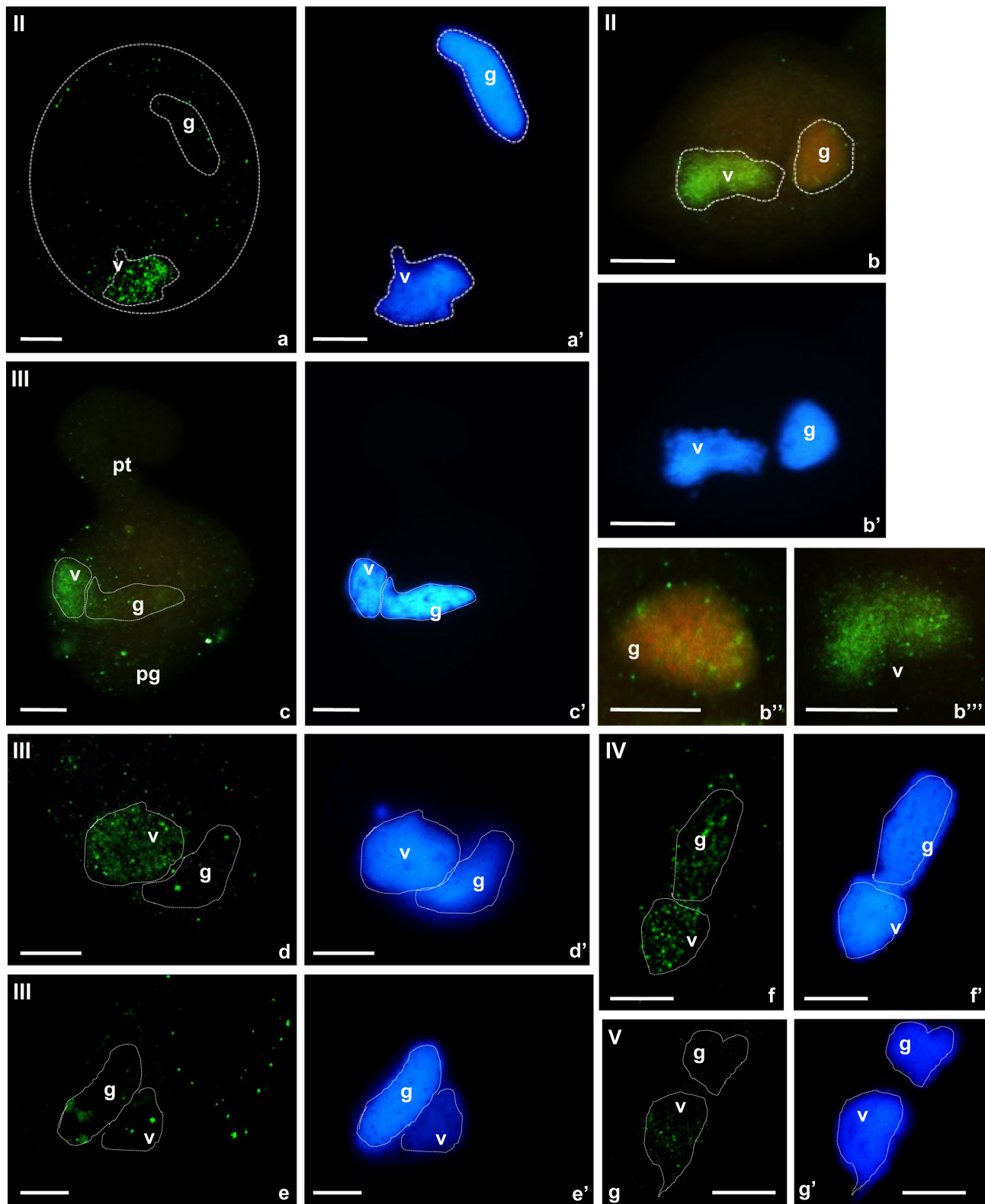



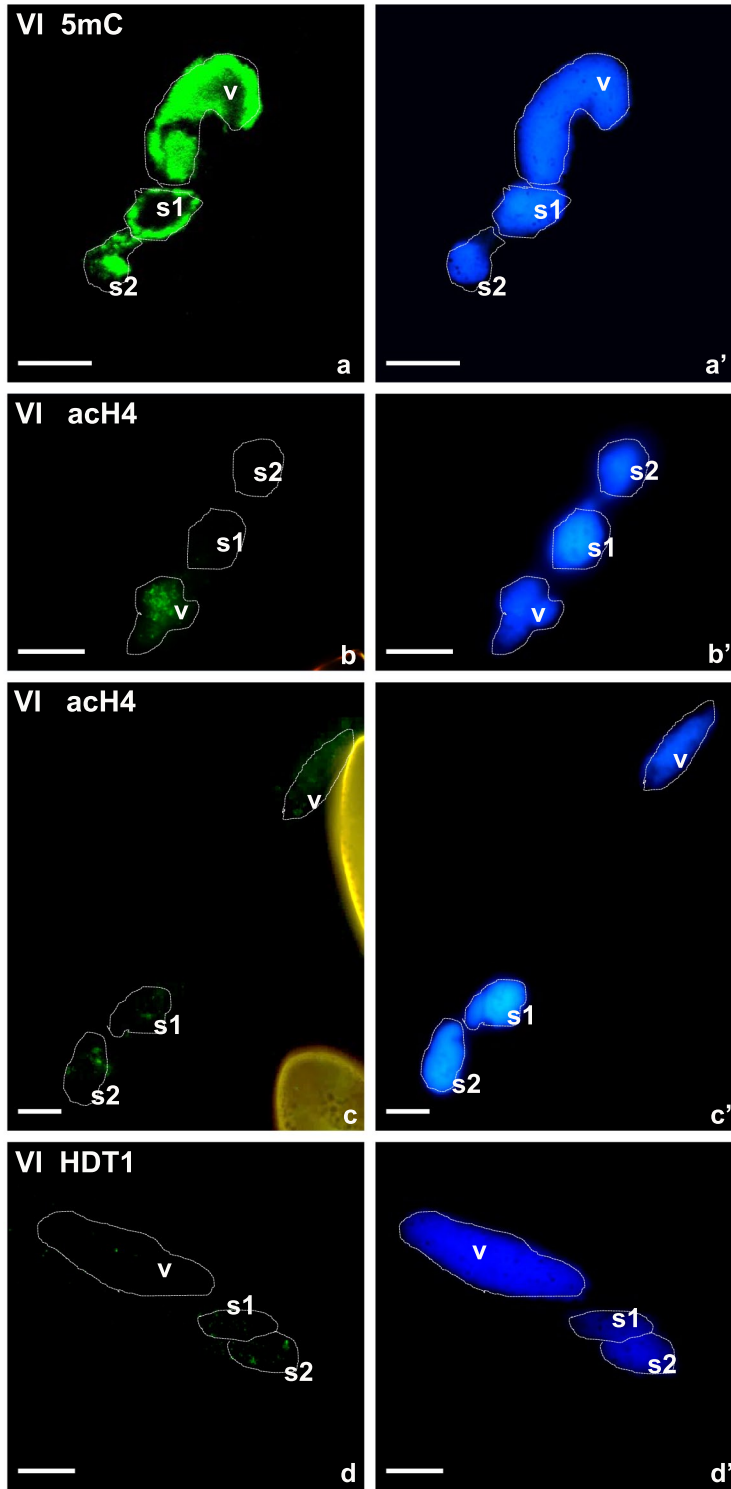

Supplement: Supplementary file 2 — Supplementary material 2 (PDF 51317 kb) [file 497_2016_289_MOESM2_ESM.pdf]
