# Supplementary material for: Epigenetic marks in the Hyacinthus orientalis L. mature pollen grain and during in vitro pollen tube growth
Source: Plant Reprod. 2016 Jul 15;29:251–63. doi: 10.1007/s00497-016-0289-3 (PMC4978762; doi:10.1007/s00497-016-0289-3)
Supplement: Supplementary file 3 — Supplementary material 3 (DOCX 24 kb) [file 497_2016_289_MOESM3_ESM.docx]

Supplementary material 3. Raw data used for the quantitative analysis of the distribution of epigenetic marks (the average fluorescence intensity [au/nucleus]) in the *Hyacinthus orientalis* I-VI developmental stages of male gametophyte, *PG* pollen grain, *PT* pollen tube, *VN* vegetative nucleus, *GN* generative nucleus

**a 5metC**

| **PG**  **stage I** | | **hydrated PG**  **stage II** | | **germinating PT**  **stage III** | | **growing PT**  **(early phase)**  **stage IV** | | **growing PT**  **(late phase)**  **stage V** | | **after GN division**  **stage VI** | | |
| --- | --- | --- | --- | --- | --- | --- | --- | --- | --- | --- | --- | --- |
| VN | GN | VN | GN | VN | GN | VN | GN | VN | GN | VN | SN1 | SN2 |
| 84.69 | 129.39 | 78.24 | 142.54 | 110.21 | 148.76 | 64.21 | 109.71 | 54.06 | 131.32 | 66.21 | 100.34 | 113.28 |
| 82.71 | 121.92 | 80.95 | 140.22 | 88.59 | 148.09 | 64.72 | 111.50 | 54.78 | 132.97 | 66.93 | 101.82 | 113.63 |
| 82.67 | 138.37 | 87.98 | 130.73 | 104.50 | 114.28 | 65.28 | 112.37 | 54.88 | 134.65 | 67.66 | 103.43 | 114.44 |
| 87.33 | 139.39 | 78.40 | 132.59 | 87.91 | 134.78 | 66.01 | 114.27 | 54.95 | 136.60 | 67.99 | 105.13 | 115.71 |
| 75.52 | 137.44 | 81.44 | 156.57 | 83.66 | 129.55 | 67.31 | 116.76 | 54.77 | 139.20 | 66.99 | 106.85 | 117.57 |
| 71.92 | 131.95 | 82.88 | 151.79 | 78.99 | 121.66 | 68.83 | 126.36 | 55.01 | 141.82 | 65.89 | 108.60 | 119.81 |
| 75.70 | 132.72 | 96.59 | 123.94 | 76.26 | 114.68 | 70.60 | 107.71 | 55.25 | 144.98 | 65.01 | 110.76 | 120.13 |
| 92.97 | 140.21 | 74.94 | 123.45 | 108.89 | 122.26 | 72.65 | 106.85 | 61.41 | 148.40 | 64.93 | 113.08 | 100.75 |
| 76.96 | 132.58 | 104.55 | 148.54 | 105.20 | 115.03 | 75.50 | 115.03 | 64.43 | 150.98 | 66.01 | 115.58 | 99.86 |
| 81.73 | 130.72 | 78.99 | 136.23 | 74.94 | 127.41 | 68.30 | 114.68 | 64.21 | 148.54 | 67.79 | 115.89 | 99.65 |

**b acH4**

| **PG**  **stage I** | | **hydrated PG**  **stage II** | | **germinating PT**  **stage III** | | **growing PT**  **(early phase)**  **stage IV** | | **growing PT**  **(late phase)**  **stage V** | | **after GN division**  **stage VI** | | |
| --- | --- | --- | --- | --- | --- | --- | --- | --- | --- | --- | --- | --- |
| VN | GN | VN | GN | VN | GN | VN | GN | VN | GN | VN | SN1 | SN2 |
| 0 | 0 | 0 | 0 | 0 | 0 | 54.23 | 44.42 | 92.80 | 46.19 | 65.21 | 36.33 | 27.15 |
| 0 | 0 | 0 | 0 | 0 | 0 | 42.36 | 43.89 | 68.19 | 66.63 | 55.28 | 40.33 | 26.93 |
| 0 | 0 | 0 | 0 | 0 | 0 | 67.92 | 50.35 | 84.95 | 58.79 | 60.29 | 27.15 | 26.72 |
| 0 | 0 | 0 | 0 | 0 | 0 | 47.61 | 33.25 | 93.39 | 63.45 | 57.79 | 26.93 | 26.28 |
| 0 | 0 | 0 | 0 | 0 | 0 | 54.72 | 46.96 | 82.29 | 50.22 | 65.35 | 26.72 | 24.31 |
| 0 | 0 | 0 | 0 | 0 | 0 | 46.19 | 38.19 | 84.95 | 63.02 | 74.95 | 26.28 | 38.56 |
| 0 | 0 | 0 | 0 | 0 | 0 | 66.63 | 31.53 | 72.32 | 62.52 | 53.07 | 42.07 | 24.31 |
| 0 | 0 | 0 | 0 | 0 | 0 | 57.33 | 29.17 | 73.53 | 56.98 | 47.73 | 44.07 | 39.21 |
| 0 | 0 | 0 | 0 | 0 | 0 | 53.18 | 35.76 | 71.18 | 47.26 | 77.24 | 47.73 | 45.22 |
| 0 | 0 | 0 | 0 | 0 | 0 | 49.41 | 43.99 | 69.21 | 52.94 | 54.47 | 28.65 | 28.93 |

**c HDT1**

| **PG**  **stage I** | | **hydrated PG**  **stage II** | | **germinating PT**  **stage III** | | **growing PT**  **(early phase)**  **stage IV** | | **growing PT**  **(late phase)**  **stage V** | | **after GN division**  **stage VI** | | |
| --- | --- | --- | --- | --- | --- | --- | --- | --- | --- | --- | --- | --- |
| VN | GN | VN | GN | VN | GN | VN | GN | VN | GN | VN | SN1 | SN2 |
| 0 | 0 | 37.32 | 0 | 37.96 | 0 | 41.49 | 29.72 | 48.13 | 47.87 | 26.39 | 26.52 | 21.35 |
| 0 | 0 | 37.96 | 0 | 39.43 | 0 | 42.49 | 30.14 | 48.24 | 48.19 | 26.45 | 21.09 | 19.13 |
| 0 | 0 | 38.03 | 0 | 37.84 | 0 | 39.43 | 30.14 | 48.39 | 47.96 | 26.49 | 26.49 | 20.70 |
| 0 | 0 | 38.44 | 0 | 38.03 | 0 | 40.86 | 29.35 | 44.24 | 47.89 | 26.58 | 19.94 | 26.42 |
| 0 | 0 | 37.43 | 0 | 37.76 | 0 | 38.44 | 32.88 | 45.34 | 45.24 | 26.25 | 26.93 | 21.03 |
| 0 | 0 | 32.89 | 0 | 36.96 | 0 | 44.63 | 27.03 | 48.28 | 43.55 | 29.76 | 24.31 | 19.93 |
| 0 | 0 | 29.99 | 0 | 36.98 | 0 | 43.76 | 32.48 | 44.76 | 45.17 | 28.82 | 26.28 | 26.22 |
| 0 | 0 | 30.21 | 0 | 29.76 | 0 | 42.36 | 28.83 | 49.02 | 41.77 | 24.94 | 21.33 | 26.56 |
| 0 | 0 | 30.87 | 0 | 28.83 | 0 | 44.64 | 29.74 | 46.82 | 39.79 | 21.39 | 20.68 | 24.31 |
| 0 | 0 | 32.41 | 0 | 37.32 | 0 | 43.82 | 27.39 | 48.51 | 38.53 | 21.34 | 21.24 | 26.28 |
